# One heart, seven leads: A case report of complex cardiac device therapy in a patient with chemotherapy-induced toxic cardiomyopathy

**DOI:** 10.1016/j.hrcr.2025.01.012

**Published:** 2025-01-31

**Authors:** Paulina Jankowska, Marwin Bannehr, Christian Georgi, Martin Seifert, Christian Butter

**Affiliations:** Department of Cardiology, University Hospital Heart Center Brandenburg, Brandenburg Medical School Theodor Fontane, Neuruppin, Germany; Faculty of Health Sciences Brandenburg, Neuruppin, Germany

**Keywords:** Device infection, Lead extraction, Wearables, Device–device interactions, Subcutaneous ICD, Cardiac resynchronization therapy, Epicardial pacemaker

**Key Teaching Points**

• Device therapy requires close monitoring due to the potential of serious complications including pacemaker pocket infections, systemic infections, and lead dysfunction.
• Better tools for screening patients who will benefit from cardiac resynchronization therapy are needed to identify nonresponders at an early stage and avoid complications.
• The changing clinical situation and complication management may require a combination of multiple implantable devices.
• Simultaneous use of a subcutaneous implantable cardioverter-defibrillator and an epicardial/transvenous cardiac resynchronization therapy pacemaker seems to be possible and safe, but programming of the devices requires particular precaution.
• Lateral position of the SQ electrode in a subcutaneous implantable cardioverter-defibrillator can lead to oversensing. In such cases, a change in programming or reposition of the SQ electrode may be necessary. Diagnostic imaging should be considered.

## Introduction

Device therapy has a well-established position in the treatment of various arrhythmias—saving lives and improving quality of life. Devices can be divided into groups based on access (eg, leadless, transvenous, subcutaneous, and wearables) and function (eg, pacemakers, defibrillators, and cardiac resynchronization therapy) of the device.

Device therapy requires close monitoring due to the potential for serious adverse events. Pacemaker pocket infections and systemic infections are among the most common complications,^1^ with risk of infection increasing with each intervention involving the pacemaker pocket. In case of a systemic infection, lead extraction must immediately be considered.

**Figure 1:**
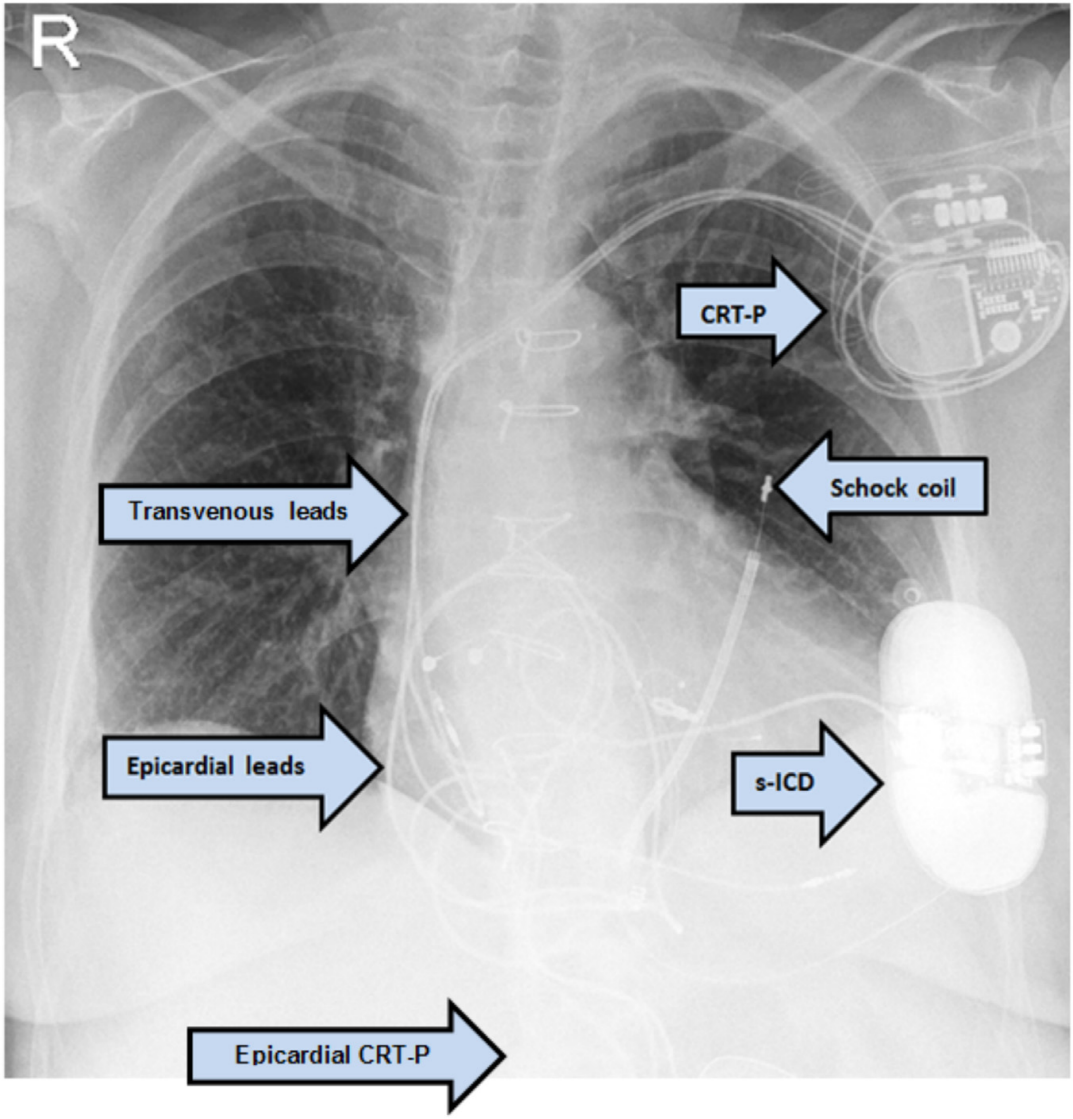
The patient's most recent chest radiograph, posteroanterior view.

Complication management and comorbidities may require innovative solutions, such as simultaneous multiple-device therapy (Figure 1). Hence, the risk for device–device interactions increases. Data regarding a combination of a subcutaneous implantable cardioverter-defibrillator (s-ICD) and an epicardial cardiac resynchronization therapy pacemaker (CRT-P) are still limited to case reports describing potential problems in programming.^2^^,^^3^ Given the increasing popularity of s-ICDs, we can expect more cases in which this combination could be useful. In the EFFORTLESS registry that examined the safety of s-ICDs, none of the 944 enrolled patients had an epicardial pacemaker and only 3% had a transvenous one.^4^

## Case report

We present the case of a 57-year-old female patient with a complete left bundle branch block and chronic heart failure with severely impaired left ventricular ejection fraction due to drug-induced toxic cardiomyopathy after she had undergone radiochemotherapy and stem cell transplantation 20 years earlier for natural killer/T-cell lymphoma. Her immune system was additionally impaired by insulin-dependent diabetes mellitus and psoriatic arthropathy with long-term prednisolone therapy. A transvenous cardiac resynchronization therapy defibrillator (CRT-D) implantation 4 years earlier was unsuccessful because of the angular course of the coronary sinus, so the left ventricular lead was placed epicardially via an anterolateral minithoracotomy. One year after the implantation, an exit block of the coronary sinus lead occurred and since then the device was programmed like a dual-chamber ICD VVI 40 beats/min.

The patient was admitted to our clinic with third-degree atrioventricular block, a fever of 38.5°C, and a perforated CRT-D pocket. The bradycardia and increased ventricular extrasystole load led to torsade de pointes, which quickly degenerated into VF; the patient experienced her first ICD shock therapy.

CRT-D interrogation showed a known exit block of epicardial left ventricular (LV) lead and, in addition to the ventricular fibrillation (VF) episode mentioned, recurrent nonsustained ventricular tachycardias. Amiodarone was introduced and a reprogramming to DDD 60 beats/min was performed. With echocardiographic evidence of vegetation on the atrial lead 4 × 5 mm and *Staphylococcus haemolyticus* in the blood cultures, antibiotic treatment of the endoplastitis with rifampicin, flucloxacillin, and gentamicin was initiated. The antibiotic treatment was de-escalated to flucloxacillin in a dose adapted to renal function according to the resistogram.

**Figure 2:**
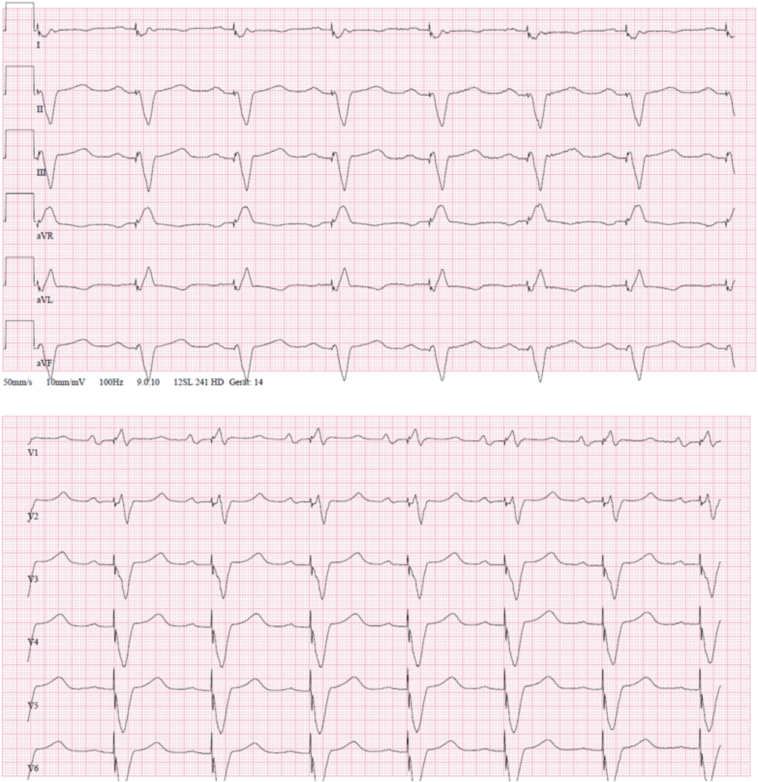
Patient’s electrocardiogram under cardiac resynchronization therapy stimulation.

Subsequently, all endovascular leads were completely explanted using excimer lasers without complications and an epicardial CRT-P was implanted during the same procedure. An electrocardiogram after CRT-P implantation showed acceptable QRS duration and morphology (Figure 2). The patient’s condition improved quickly, and they were discharged with a wearable defibrillator after treatment of acute decompensated heart failure with intravenous loop diuretic and antibiotic therapy.

During follow-up, VF recurred and was terminated by shock delivery from the wearable defibrillator. In addition, the epicardial CRT-P system started showing an increase in the stimulation threshold of the bipolar LV lead and the output parameters needed to be adjusted.

After transesophageal echocardiography, sterile blood cultures and positive screening, an elective implantation of an s-ICD was performed 3 months later. The SQ electrode was inserted laterally because of sternal cerclage on the left side. Defibrillator threshold test proved appropriate detection and successful VF termination in intraprocedural testing. The QRS paced by the epicardial CRT-P was sensed correctly by the s-ICD.

The patient was hospitalized 2 more times in the following months for mitral valve clipping, recompensation, and cavotricuspid isthmus ablation of atrial flutter.

During the follow-up examinations, a further critical increase in the stimulation thresholds of the LV and also right ventricular leads was noticed. On phlebography, both subclavian veins and the superior vena cava were open. Given these findings, we decided to reimplant a transvenous CRT-P. The procedure was performed without complications and the LV lead could be implanted successfully transvenously this time.

**Figure 3:**
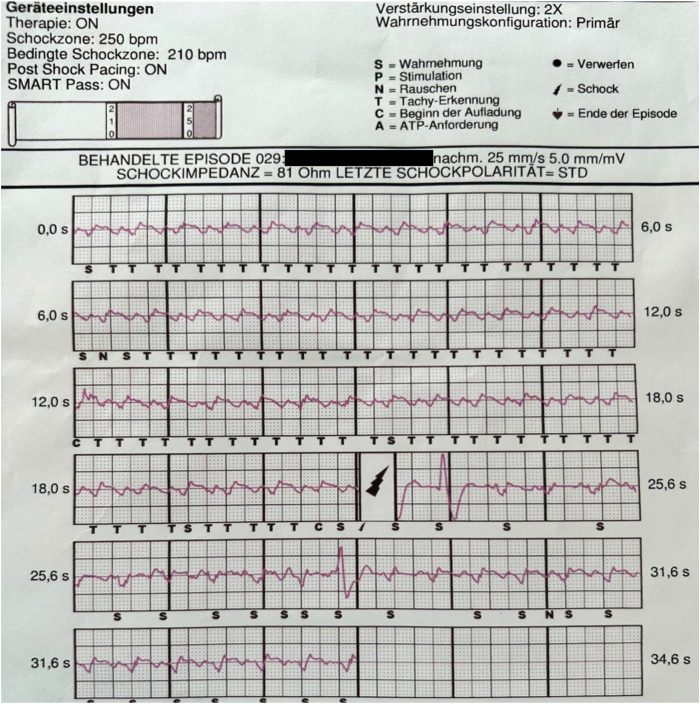
Inadequate shock due to oversensing of the subcutaneous implantable cardioverter-defibrillator.

A few months later, the patient was hospitalized again due to inadequate shock because of oversensing of the s-ICD due to double counting of the T-wave during ventricular stimulation of the biventricular pacemaker (Figure 3). The perception vector was reprogrammed to secondary, with double amplification. After this change in the follow-up of 8 months in programming, no further inadequate s-ICD therapies occurred. The patient’s condition is stable, but they suffer from exhaustion due to numerous surgeries and hospitalizations. Left ventricular ejection fraction remained severely impaired (15%), which lowered the quality of life and worsened the prognosis, but did not break the patient’s strong will to live. Currently the patient is registered for a heart transplantation.

## Discussion

This case showed that device therapies require innovative solutions, close monitoring, and an individualized approach. Complex device therapy, including simultaneous use of an s-ICD and an epicardial or a transvenous CRT, seems to be possible, as long as screening, implantation, and programming take place in a specialized center.

Through s-ICD screening among patients, who have had another device implanted, sensing of paced QRS can be controlled; thus reducing the risk of inadequate therapies. Also the anatomic position of both already existing and planned leads needs to be considered. In this case, we cannot exclude that the lateral position of the SQ electrode may have contributed to the oversensing problem of the s-ICD. A few case reports can be found in the literature in which the SQ electrode had to be placed in a different position^5^^,^^6^ due to increased defibrillation threshold or inadequate shocks. In our case, however, the change in programming sufficed to improve sensing. Should the oversensing reappear, then computed tomography would be recommended to better access the position of the SQ lead.

The programming may be challenging as possible device–device interactions need to be taken into consideration. In our patient, all leads of both CRT systems were programmed bipolar. A few available case reports recommended avoiding unipolar configuration of the pacemaker while implanting s-ICD to avoid oversensing.^7^, ^8^, ^9^, ^10^ Another way to reduce the risk of double counting and thus inadequate shocks is programming of the VF detection above the double of the upper pacing rate. This option, however, is reserved for older and less active patients, as the sensor of the pacemaker would have to be lowered to heart frequency of a maximum of 100–110 beats/min.

Keeping the number of necessary surgeries as low as possible, reduced risk of infection or other complications and is crucial from the patient's perspective. That is the reason multifunctional devices—like CRT-D systems—appear to have great potential, as the clinical situation may rapidly change. In an observational cohort study, 6.7% of patients with single-chamber ICD required >5% pacing while programmed at VVI 40 beats/min.^11^ In another study, 5% of the patients with a pacemaker required an ICD upgrade.^12^ The s-ICDs offer protection against dangerous arrhythmias and have a low risk of infection, but currently cannot deliver antibradycardia pacing. In cases of an atrioventricular block, primary application of conduction system pacing could prevent pacing-induced cardiomyopathy and CRT upgrade.

The numerous complications of the device therapy that occurred in this case, including endoplastitis, recurring electrode dysfunctions, and inadequate shock, emphasized the importance of accurate indication and close monitoring.^13^ Although the indication for the resynchronization therapy was correct according to guidelines, our patient’s left ventricular ejection fraction remained severely impaired, which indicates that better tools for screening patients who will benefit from CRT therapy are needed to identify nonresponder at an early stage. In this case, complications started with the first CRT-D implantation, which required anterolateral minithoracotomy. Four years later, the coronary sinus lead insertion was possible transvenously in our clinic without problems. It allows us to assume the benefit of treatment in more specialized centers with greater experience.

The case highlighted the importance of constant monitoring of high-risk patients. In this case, the patient survived the VF without neurologic deficits, thanks to the bridging with a wearable defibrillator.

Finally, we see how much an individualized approach and shared decision making are crucial in the most challenging cases. With the right guidance and support, our patient was able to make it through multiple complications and remained motivated to try further therapeutic options, fighting for years of life and a better quality of life. This certainly would not be a solution for every patient, which is why detailed discussion about therapy options, their chances of success, and possible complications will remain the foundation of every therapy.

## Conclusion

Device therapy requires constant monitoring due to possible complications, especially systemic infections. An extravascular device is an alternative for patients with recurrent device infections. Complex device therapy with an s-ICD and a transvenous/epicardial CRT-P is possible, but the programming may be challenging due to the risk of (over)sensing problems and resulting inadequate shocks. Better tools for screening patients who will benefit from CRT therapy are needed.

## Disclosures

The authors have no conflicts of interest to disclose.
